# Two-Level Spatio-Temporal Feature Fused Two-Stream Network for Micro-Expression Recognition

**DOI:** 10.3390/s24051574

**Published:** 2024-02-29

**Authors:** Zebiao Wang, Mingyu Yang, Qingbin Jiao, Liang Xu, Bing Han, Yuhang Li, Xin Tan

**Affiliations:** 1 Changchun Institute of Optics, Fine Mechanics and Physics, Chinese Academy of Sciences, Changchun 130033, China; wangzebiao21@mails.ucas.ac.cn (Z.W.); voynichjqb@163.com (Q.J.); xuliang_998@163.com (L.X.); hanbing@ciomp.ac.cn (B.H.); liyuhang192@mails.ucas.ac.cn (Y.L.); 2 University of Chinese Academy of Sciences, Beijing 100049, China

**Keywords:** micro-expression recognition, two-stream architecture, feature fusion algorithm, shifted window-based self-attention

## Abstract

Micro-expressions, which are spontaneous and difficult to suppress, reveal a person’s true emotions. They are characterized by short duration and low intensity, making the task of micro-expression recognition challenging in the field of emotion computing. In recent years, deep learning-based feature extraction and fusion techniques have been widely used for micro-expression recognition, particularly methods based on Vision Transformer that have gained popularity. However, the Vision Transformer-based architecture used in micro-expression recognition involves a significant amount of invalid computation. Additionally, in the traditional two-stream architecture, although separate streams are combined through late fusion, only the output features from the deepest level of the network are utilized for classification, thus limiting the network’s ability to capture subtle details due to the lack of fine-grained information. To address these issues, we propose a new two-level spatio-temporal feature fused with a two-stream architecture. This architecture includes a spatial encoder (modified ResNet) for learning texture features of the face, a temporal encoder (Swin Transformer) for learning facial muscle motor features, a feature fusion algorithm for integrating multi-level spatio-temporal features, a classification head, and a weighted average operator for temporal aggregation. The two-stream architecture has the advantage of extracting richer features compared to the single-stream architecture, leading to improved performance. The shifted window scheme of Swin Transformer restricts self-attention computation to non-overlapping local windows and allows cross-window connections, significantly improving the performance and reducing the computation compared to Vision Transformer. Moreover, the modified ResNet is computationally less intensive. Our proposed feature fusion algorithm leverages the similarity in output feature shapes at each stage of the two streams, enabling the effective fusion of multi-level spatio-temporal features. This algorithm results in an improvement of approximately 4% in both the F1 score and the UAR. Comprehensive evaluations conducted on three widely used spontaneous micro-expression datasets (SMIC-HS, CASME II, and SAMM) consistently demonstrate the superiority of our approach over comparative methods. Notably, our approach achieves a UAR exceeding 0.905 on CASME II, making it one of the few frameworks in the published micro-expression recognition literature to achieve such high performance.

## 1. Introduction

Facial expressions, along with voice, speech, hand gestures, and body posture, play a crucial role in conveying signals about an individual’s emotional state. Non-verbal cues, including facial expressions, carry a wealth of information and can consciously or unconsciously communicate human emotions. They serve as a vital source of information and play a significant role in emotion analysis [1]. Many psychologists argue that while individuals may use different languages to communicate based on their cultural contexts, the expression of emotions is largely universal [2].

Macro-expressions are spontaneous and typically last between 0.5 and 4 s. They involve underlying facial movements that cover a large area of the face. In contrast, micro-expressions (ME) are unconscious, rapid, and localized expressions with a shorter duration, ranging from 0.065 to 0.5 s [3]. Macro-expressions can be consciously suppressed by individuals to mask their true emotions. However, MEs are spontaneous and cannot be easily faked or suppressed. As a result, they have the potential to reveal genuine emotions. This unique characteristic of MEs has led to an increasing demand for micro-expression recognition (MER) technology [4].

The first spontaneous MER research can be traced to Pfister et al.’s work [5], which utilized a Local Binary Pattern from Three Orthogonal Planes (LBP-TOP) on the first public spontaneous ME dataset: SMIC [6]. Following the work of [6], various approaches based on appearance and geometry features [7,8] were proposed to improve the performance of MER.

**Figure 1:**
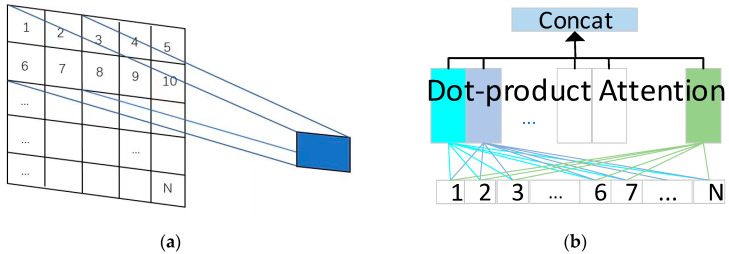
Comparison of the different feature extraction methods of CNN and transformer. (**a**) Convolutional Kernel; (**b**) Multi-head Self-attention Mechanism.

Early work by and large uses the Convolutional Kernel [8,9,10] to extract information from ME video frames. This kind of pixel-level operator can be considered to capture local spatial relations. Graph Convolutional Network (GCN)-based architectures [11,12] have also been used to learn global relationships between different spatial regions. However, they can only learn global spatial relations after local features are extracted. While the movement of adjacent local facial muscles is closely related, it is important to automatically capture the local and global relations of the adjacent area in optical flow at the same time. The analysis of optical flow allows for capturing the weak movement trend of muscles between frames. As shown in Figure 1 [2], the relations between block 1 and N will hardly ever be learned by Convolutional Neural Network (CNN) but have been considered at the beginning of the Multi-head Self-attention Mechanism (MSM). MSM-based networks are called Transformers. The above describes the advantages of MSM compared with Convolutional Kernel, but MSM is more computational than Convolutional Kernel. Vision Transformer (ViT) based architecture has also been used to capture the local and global relationships of optical flow jointly [2]. Nevertheless, it is important to consider that the motion correlation between distant facial muscles tends to be weak. ViT calculates the relationship between all local spaces in the optical flow, resulting in a considerable number of unnecessary calculations being introduced.

As a classical architecture in the field of video understanding, two-stream architecture was first proposed in [13] and used for MER in [14], in which the two separate streams are implemented by CNN and combined by late fusion. Late fusion refers to the process where two stream networks generate category scores independently and then combine these scores. However, this fusion approach does not allow for multi-level fusion of the features extracted by the two encoders, which may limit the network’s ability to capture subtle details, especially in the case of weak MEs. This limitation results in a lack of fine-grained information. Therefore, the traditional two-stream architecture may not be optimal for capturing subtle variations in MEs, which are crucial for accurate recognition.

To achieve optimal MER performance with limited ME data, our deep learning MER architecture implements a two-stream architecture with two inputs. The first input is a single frame containing the spatial information of the video, while the second input is the interframe optical flow containing the temporal information of the video. To capture the local and global relations of the adjacent area in optical flow simultaneously and minimize computation, our temporal encoder uses the Swin Transformer architecture [15], which is based on MSM. This hierarchical transformer uses shifted windows to compute its representation, enabling self-attention computation to be restricted to non-overlapping local windows while allowing cross-window connections. As a result, it can automatically capture the local and global relationships of different regions in a single window simultaneously. The Swin Transformer has linear computational complexity with respect to image size, whereas the computational complexity of ViT is quadratic with respect to the image size. The primary function of the spatial encoder is to extract static face texture features. Therefore, the global relationship between different spatial regions can be ignored to reduce computation. For this purpose, our spatial encoder adopts the ResNet architecture [16], which is based on a Convolutional Kernel. Furthermore, to match the subsequent feature fusion algorithm, the layers of each stage of the ResNet architecture are adjusted to be consistent with each stage of the Swin Transformer. This modified ResNet architecture is named ResNet13, according to the number of convolutional layers.

In MER networks employing a two-stream architecture, the features utilized for classification are obtained by combining the output features from the final level of the two encoders. It is crucial to incorporate low-level features that capture fine-grained information from images since MEs are subtle and require detailed analysis. These low-level features are derived from the shallow layers of the network. To effectively fuse the multi-level spatio-temporal features, we propose a feature fusion algorithm. The features used for classification are generated by combining the output features from different stages of the two encoders.

In this work, we show how a transformer-based deep learning architecture and a CNN-based deep learning architecture can be applied to MER in a way that is superior to the comparative approaches. The main contributions of the present work are as follows:

(1) We propose a novel spatio-temporal deep learning architecture for video-based MER, taking full advantage of transformer and CNN. We name it a Two-Level Spatio-Temporal Feature Fused Two-Stream Network (TFT). To the best of our knowledge, ours is the first deep-learning MER work utilizing the transformer-based Swin Transformer architecture and the CNN-based ResNet architecture at the same time.
(2) We propose a feature fusion algorithm, which fuses the output features of different stages of the two encoders to achieve a multi-level fusion of spatio-temporal features.
(3) The frames near the apex frame are selected for data enhancement in the sequence after temporal interpolation. The performance of mean, weighted average, and LSTM (long short-term memory) aggregators are also analyzed.

The paper is organized as follows. Some related work of the MER and the architecture we used are reviewed in Section 2. In Section 3, the proposed approach with TFT and complementary approaches are introduced. The results of the three well-known and popular ME datasets are given in Section 4. The TFT architecture is discussed in Section 5. Finally, a conclusion is presented in Section 6.

## 2. Related Works

### 2.1. MER

Since the release of SMIC in 2013, the number of studies on automatic MER has steadily increased. From the handcrafted computer vision approaches to more recent deep learning approaches. Important components of the deep learning-based MER approach include inputs and networks [17].

#### 2.1.1. Inputs

Due to the subtle motion and limited sample size of ME sequences, different inputs have a great impact on the performance of MER. Pre-processing involving face detection, alignment, motion magnification, temporal normalization, and regions of interest division has been undertaken to improve MER performance.

There has been a lot of research [18,19] conducted on static images without time information due to the availability of a large number of face images on the network and the development of face expression recognition technology. Researchers have explored MER based on the apex frame with the highest intensity of facial motion of all frames and found that deep learning under the single apex frame can achieve good performance. Additionally, it has been shown that the apex frame-based approach can effectively utilize the massive static images in macro-expression databases and obtain better performance than onset-apex offset sequences or the entire video [20].

Temporal dynamics along video sequences are critical to improve MER performance, as facial movements are subtle in the spatial domain and change quickly in the temporal domain. Architectures based on 3D CNN and Recurrent Neural Networks have been developed for MER [8,21], and it has been demonstrated that onset, apex, and offset frames can provide enough spatial and temporal information for MER. Dynamic images generated by using a rank pooling algorithm [22], active images obtained by estimating and accumulating the changes of each pixel component [23], and optical flow [24] all encapsulate the temporal information of video sequences and have been successfully applied to MER.

Given the advantages of apex frames and dynamic image sequences, some research works have analyzed multiple inputs to learn features from different cues in ME videos. For example, in Liu et al.’s work [25], apex frames and optical flows were used to extract static spatial and dynamic temporal features, and local face areas of the apex frame were added as input to increase the robustness of MER. Additionally, Sun et al. [26] fully mined the time information by using optical flow and sequence.

#### 2.1.2. Deep Networks

The utilization and design of efficient network blocks addressed two major challenges in ME research: overfitting on small-scale ME datasets and the low intensity of MEs. Various studies [27] used residual blocks for the robust identification of small-scale ME datasets. Additionally, Inception, Capsule Neural Network, and non-local network structures were adopted to improve the efficiency of MER, with global spatial and temporal attention modules and adaptive feature channel weighting also being explored [28]. Several studies have combined multi-stream and cascaded structures to further investigate multi-view sequence information.

### 2.2. Swin Transformer

CNNs have long been the cornerstone of most deep-learning algorithms in computer vision. However, convolution operates on a small fixed-size window, making it unable to extract remote relationships. Transformers rely on MSM to learn relationships between sequence elements and capture “long-term” dependencies. In early Transformer-based models, tokens are all of a fixed scale, which becomes intractable for high-resolution images due to the quadratic computational complexity of self-attention.

To resolve these challenges, the Swin Transformer [15] creates hierarchical feature maps with linear computational complexity relative to image size. It forms a hierarchical representation by beginning with small-sized patches and progressively merging neighboring patches in deeper Transformer layers.

**Figure 2:**
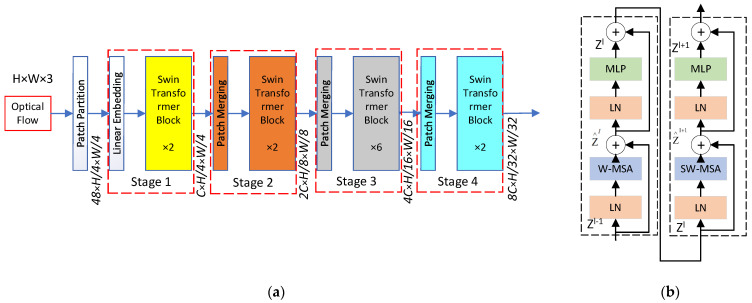
(**a**) The architecture of the Swin Transformer; (**b**) Two successive Swin Transformer Blocks. W-MSA and SW-MSA are multi-head self-attention modules with regular and shifted windowing configurations, respectively.

The input optical flow is first divided into non-overlapping patches by a patch-splitting module, similar to ViT. Each patch is considered a “token” with features formed by concatenating the original pixel’s three-channel values. The implementation details can be found in [15]. An overview of the Swin Transformer architecture is depicted in Figure 2a [15]. The detail of a Swin Transformer Block is shown in Figure 2b [15].

### 2.3. ResNet

With the development of deep learning, it is gradually realized that increasing the width and depth of the network can improve the performance of the network. However, many studies have shown that the deeper the network layer, the more difficult it is to train. This is because of the chain rule in the backpropagation algorithm, if the gradient of each layer is between (0, 1) and shrinks layer by layer, then the gradient will disappear. Conversely, if the gradient transmitted layer by layer is greater than 1, then after layer-by-layer expansion, there will be a gradient explosion.

**Figure 3:**
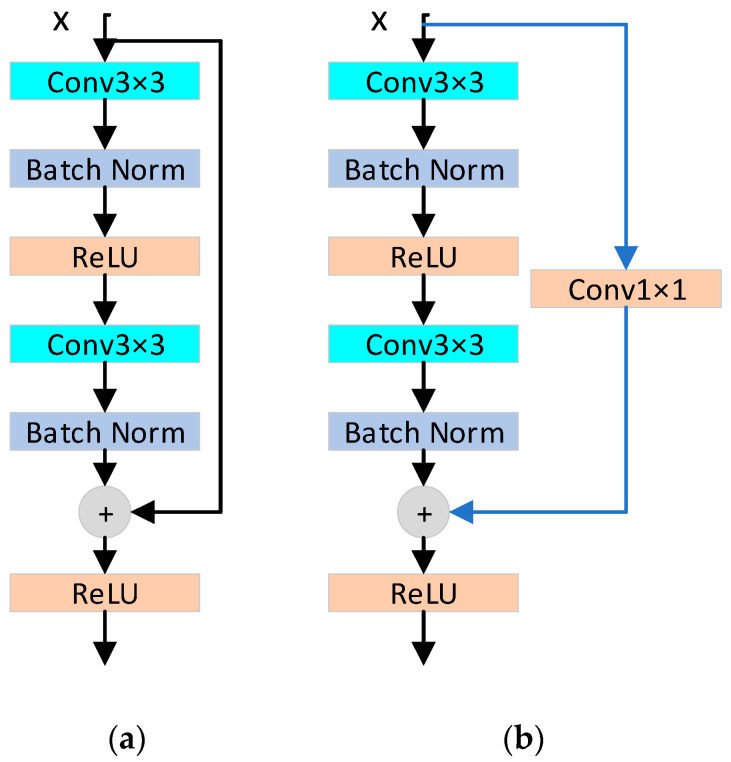
The residual basic block of ResNet. (**a**) The dimensions of input feature and output feature are consistent; (**b**) The dimensions of input feature and output feature are different.

Although the problem can be solved by means of data regularization and initialization, deepening the depth of the network when the network converges does not improve the accuracy of the training set, but presents a saturated or decreased state, which leads to the “degradation” of the network. Aiming at the “degradation” problem of network training, He Keming et al. [16] proposed an advanced neural network in 2015, namely residual neural network (ResNet). Figure 3 [16] shows the residual basic block.

Unlike previous methods of network connection, ResNet introduces a skip connection (also known as a residual connection) that allows several weight layers to be skipped. Residuals connect shallow and deep layers, and gradients from deep layers can be transferred directly to shallow layers without additional parameters and computational complexity. ResNet avoids the phenomenon of gradient disappearance in the normal backpropagation from the deep layer to the shallow layer and provides a good solution to the problem of network degradation.

### 2.4. Long Short-Term Memory (LSTM)

Long Short-Term Memory Network (LSTM) [29] is an improved recurrent neural network that solves the problem of it that cannot handle long-distance dependencies.

The computation details of each layer are:

(1) $$t=1\ldots F,l=L_{T}+1\ldots L_{A}$$

(2) $$f_{t}=\sigma (W_{f}\cdot \left[Z_{l}^{t-1},Z_{l-1}^{t}\right]+b_{f})$$

(3) $$i_{t}=\sigma (W_{i}\cdot \left[Z_{l}^{t-1},Z_{l-1}^{t}\right]+b_{i})$$

(4) $$O_{t}=\sigma (W_{o}\cdot \left[Z_{l}^{t-1},Z_{l-1}^{t}\right]+b_{o})$$

(5) $$C_{t}^{\text{’}}=tanh(W_{C}\cdot \left[Z_{l}^{t-1},Z_{l-1}^{t}\right]+b_{C})$$

(6) $$C_{t}=f_{t}\times C_{t-1}+i_{t}\times C_{t}^{\prime }$$

(7) $$Z_{l}^{t}=O_{t}+tanh(C_{t})$$

**Figure 4:**
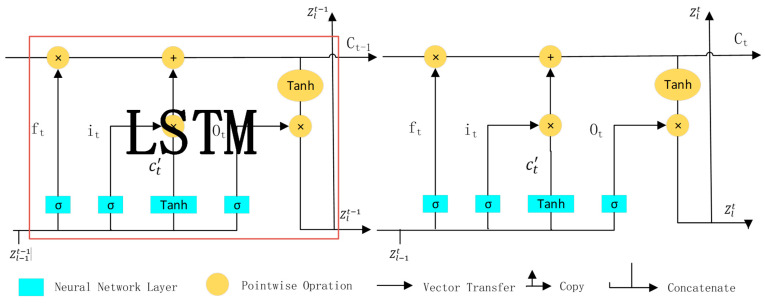
The repeating module in an LSTM aggregator layer.

In the given equations, *F* represents the chosen number of frames in each video sample, L_A_ stands for the total number of layers. $Z_{l}^{t\;}$ represents the outputs of layer l after t frames have been processed. σ(z) indicates the Sigmoid activation function, tanh⁡(z) represents the tanh activation function. *W* and *b* represent weight and bias, respectively. [*z*1, *z*2] indicates concatenate *z*1 and *z*2. Once all frames have been processed in this manner, the result is a single feature set that describes the entire ME video sample. These features are then input into the classification head for the ultimate MER classification. Details on how the previous output joins the latter training are presented in Figure 4 [2].

### 2.5. Adadelta Optimizer [30]

Compared with the gradient descent, the update rule of Adagrad does not set a fixed value for the learning rate, and different learning rates are used for each parameter optimization during each iteration. Suppose at time *t* of a certain iteration, the gradient of the objective function to the parameters is as follows:

(8) $$g_{t,i}=\nabla_{\theta }J(\theta_{i})$$

where *J* represents the objective function, *θ* represents the parameter, and ∇ represents the derivative sign.

Ordinary stochastic gradient descent algorithm uses the same learning rate for all $\theta_{i}$. Therefore, when iterating to the t time, the change process of a certain parameter vector $\theta_{i}$ is as follows:

(9) $$\theta_{t+1,i}=\theta_{t,i}-\eta \cdot g_{t,i}$$

(10) $$∆\theta_{t}=-\eta \cdot g_{t,i}$$

where η is the learning rate, $∆\theta_{t}$ represents the change in the parameter $\theta_{i}$ at the *t* iteration.

In Adagrad’s update rule, the learning rate will change with each iteration according to the historical gradient:

(11) $$\theta_{t+1,i}=\theta_{t,i}-\frac{\eta }{\sqrt{G_{t}+\varepsilon }}\cdot g_{t,i}$$

(12) $$∆\theta_{t}=-\frac{\eta }{\sqrt{G_{t}+\varepsilon }}\cdot g_{t,i}$$

where $G^{t}\in R^{d\times d}$ is a diagonal matrix, For each diagonal position *i*, the value of *i* accumulates to the sum of the squares of the gradient of the corresponding parameter *θ_i_* for *t* iterations; ε is a smooth entry that prevents zero division and the value is generally 1 × 10^−8^.

Adagrad calculates the sum of the squares of the gradient in the denominator because all the parameters must be positive so that the cumulative sum of the denominator will become larger and larger during the training process. In this way, in the later stage of learning, the updating ability of the network will become weaker and weaker, and the ability to learn more knowledge will also become weaker and weaker because the learning rate will become extremely small. In order to solve this problem, the Adadelta algorithm is proposed. The gradient sum is recursively defined as the decay mean of the historical gradient squared. The dynamic mean value depends only on the current gradient value and the mean value of the previous time:

(13) $$E{\left[g^{2}\right]}_{t}=\gamma E{\left[g^{2}\right]}_{t-1}+(1-\gamma )g_{t}^{2}$$

where *γ* is similar to the momentum term and is around 0.9.

Simply replacing the original *G_t_* as $E{\left[g^{2}\right]}_{t}$:

(14) $$∆\theta_{t}=-\frac{\eta }{\sqrt{E{\left[g^{2}\right]}_{t}+\varepsilon }}\cdot g_{t,i}$$

The denominator is simply denoted as *RMS*, representing the root mean square error of the gradient:

(15) $$∆\theta_{t}=-\frac{\eta }{RMS{\left[g\right]}_{t}}\cdot g_{t}$$

In the update, the exponential decay mean is defined instead of the gradient squared. Update with the square of the parameter:

(16) $$E{\left[{∆\theta_{t}}^{2}\right]}_{t}=\gamma E{\left[{∆\theta_{t}}^{2}\right]}_{t-1}+(1-\gamma )∆\theta_{t}$$

By substituting *RMS*[∆*θ*]*_t_*_−1_ for the learning rate *η*, the Adadelta update rule is obtained:

(17) $$\theta_{t+1}=\theta_{t}-\frac{RMS{\left[∆\theta \right]}_{t-1}}{RMS{\left[g\right]}_{t}}\cdot g_{t}$$

## 3. Proposed Method

The proposed method includes the proposed architecture, the transfer learning method, and the model optimization method used in model training.

### 3.1. Proposed Architecture

This proposed architecture includes a spatial encoder (modified ResNet) for learning texture features of the face, a temporal encoder (Swin Transformer) for learning facial muscle motor features, a feature fusion algorithm for integrating multi-level spatio-temporal features, a classification head, and a weighted average operator for temporal aggregation. The two encoders respectively serve as a stream of the two-stream architecture.

**Figure 5:**
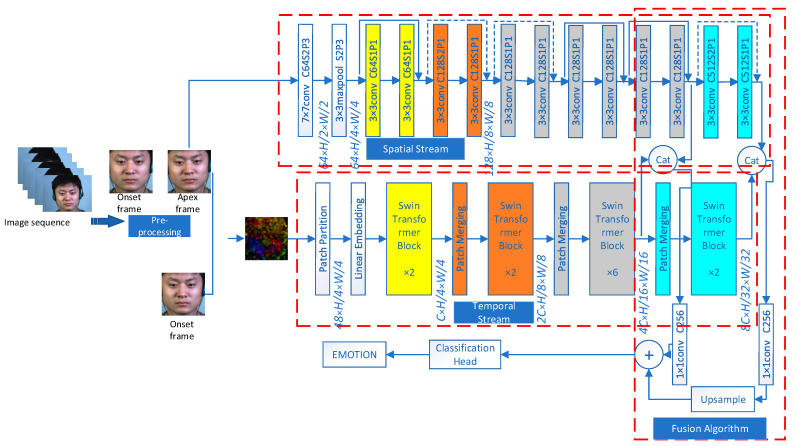
The proposed architecture.

An overview of the proposed architecture is shown in Figure 5. In the present work, we propose an architecture that takes advantage both of transformer-based Swin Transformer [15] and CNN-based ResNet [16]. The approach overcomes many weaknesses of the existing MER methods as discussed in the previous section. Importantly, our architecture is able to extract, fuse, and thus benefit from multi-level local and global spatio-temporal features.

#### 3.1.1. Two-Stream Architecture

The central concept of this architecture revolves around dividing each optical flow into a series of patches and then assigning them into non-overlapping windows, with the Swin Transformer encoder being employed to capture the distant spatial relationships between the patches within each window. By shifting these windows, the architecture is able to establish the relationships between different windows, thus obtaining time series features. Additionally, static spatial texture features are extracted from the apex frame using Convolutional Kernel.

**Figure 6:**
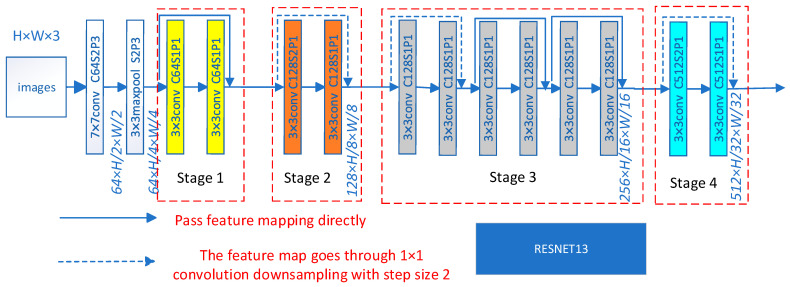
The architecture of the ResNet13.

Our spatial encoder utilizes a modified ResNet architecture, comprising a convolution layer, six residual basic blocks, and a pooling layer. The convolution layer performs feature extraction from the apex frame through convolution operation, with the four stages consisting of 64, 128, 256, and 512 Convolutional Kernels, respectively. Each stage contains 1, 1, 3, and 1 residual basic block, each comprising two 3 × 3 convolutional layers connected to batch normalization and ReLU activation functions. By adding the input features and features extracted from the input features via two convolution layers, each residual block’s output is obtained through the activation function. For inconsistent feature dimensions, we perform dimension matching on the original input features to modify the number of channels for element-wise addition. The residual block connects shallow input and deep output, preventing gradient disappearance. The pooling layer uses average pooling to downsample input features to reduce unnecessary information. Figure 6 shows the detailed structure.

The detailed architecture specifications are shown in Table 1, describing the specific parameters of the architecture shown in Figure 6, and an input image size of 224 × 224 is assumed for the architecture.

Where “K64” denotes the number of Convolutional Kernel in this layer is 64.

We adopt the Swin Transformer architecture as the temporal encoder, as described in the previous section. The detailed architecture specifications are shown in Table 2, describing the specific parameters of the architecture shown in Figure 2a.

In the architecture, the assumed input image size is 224 × 224. “Concat n × n” refers to concatenating n × n neighboring features in a patch, resulting in downsampling of the feature map by a rate of n. “96-d” denotes a linear layer with an output dimension of 96. “Win. Sz. 7 × 7” indicates a multi-head self-attention module with a window size of 7 × 7.

Experiments of ablation are designed to illustrate the effectiveness of the proposed two-stream architecture, and a full discussion is made in Section 5.

#### 3.1.2. Feature Fusion Algorithm

After extracting the temporal and spatial features from the ME video, we propose a feature fusion algorithm to combine these features before performing the final classification. Since MEs are subtle and weak, it is important to consider the low-level features that contain fine-grained information. The main concept of this algorithm is to integrate multi-level spatio-temporal features to capture the fine-grained information of MEs.

**Figure 7:**
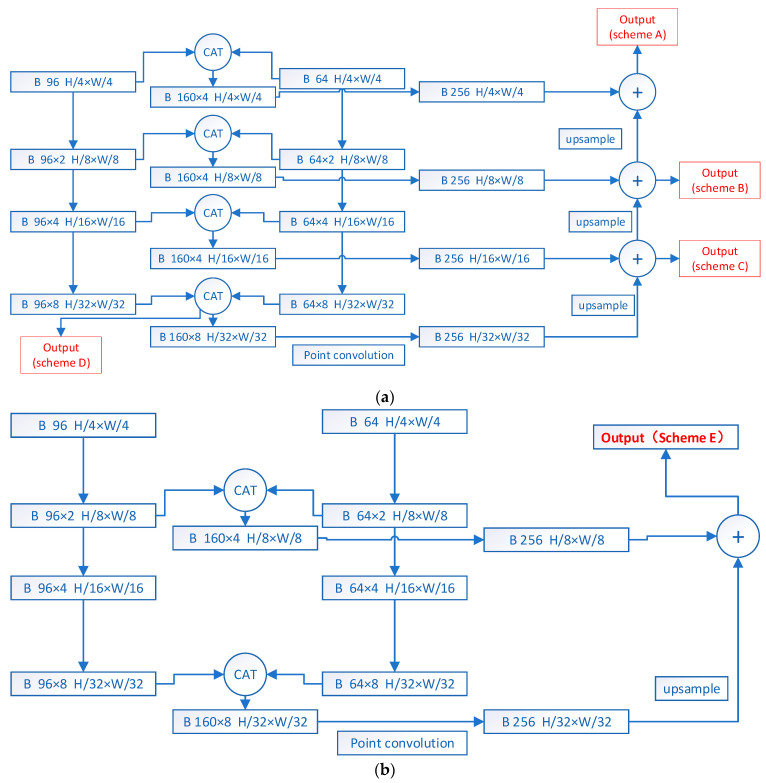
Different feature fusion schemes. (**a**) Scheme A~D; (**b**) Scheme E.

The proposed two-stream networks both have four stages. According to the different stages from which the features to be fused come, we tried five fusion schemes, as shown in Figure 7.

The fusion algorithm combines the multi-level features extracted by the spatial encoder and temporal encoder. It performs concatenation on the outputs of the same stage from both streams. Then, point convolution is applied to ensure that the number of channels in different stages is consistent. The features from the higher stage are upsampled to the same size as the features from the previous stage. The addition operation is then performed on the two features from adjacent stages. The fusion process progresses step by step from the high stage to the low stage. Scheme C has been proven to be the most effective, and thus, it is adopted as our final fusion scheme.

Taking fusion scheme C as an example, the sizes of the output features from the last two stages of the temporal stream network are B × 96 × 4 × H/16 × W/16 and B × 96 × 8 × H/32 × W/32, respectively. The sizes of the output features from the last two stages of the spatial stream network are B × 64 × 4 × H/16 × W/16 and B × 64 × 8 × H/32 × W/32, respectively. Firstly, the output features of the corresponding stages from both streams are concatenated, resulting in features of size B × 160 × 4 × H/16 × W/16 and B × 160 × 8 × H/32 × W/32 for the two stages, respectively. Then, point convolution is applied to ensure that the number of channels in the features from different stages is 256. Additionally, the output feature from the last stage is upsampled to match the size of the output feature from the previous stage. Finally, the two features are added together. Here, B represents the batch size, and H and W refer to the height and width of the input feature map, respectively.

Experiments of ablation are designed to illustrate the effectiveness of the proposed fusion algorithm, and a full discussion is made in Section 5.

#### 3.1.3. Temporal Aggregation

The proposed weighted average operator takes into account the fact that the apex frame contains the most abundant features and decreases successively among the five adjacent frames. This design is based on the observation that the feature richness of the inputs varies across the frames. By conforming to this feature distribution, the weighted average operator is able to achieve better temporal aggregation.

After the feature fusion block, our architecture includes a classification head consisting of two fully connected layers. These layers enable the final classification using the SoftMax activation function. The output size of the first fully connected layer is set to 256. A ReLU activation function is applied between the two fully connected layers, enhancing the non-linear capabilities of the model.

After the classification head, the outputs of each pair of images are used as inputs to the temporal aggregation layer. Due to the use of a high-speed camera, the selected frames are highly similar, and their relationship is weak or even chaotic. As a result, LSTM is not applicable in this scenario and would introduce unnecessary computational complexity. However, we can leverage the similarities of the selected frames to enhance the data using a weighted average operator. This helps alleviate the issue of having a small amount of ME samples. In our weighted average operator, we assign weights of 0.1, 0.2, 0.4, 0.2, and 0.1 to the selected five frames in order, taking into account the strongest features of the apex frames. This choice provides a more effective representation of the ME sequence compared to a simple mean operator. In the experiment using an LSTM aggregator, we placed three LSTM layers within the aggregation block before the classification head.

Experiments of ablation are designed to illustrate the effectiveness of the proposed weighted average operator, and a full discussion is made in Section 5.

### 3.2. Transfer Learning

The key concept behind the proposed transfer learning method is to ensure consistency in the input form and preprocessing method during both pre-training and fine-tuning processes. This helps in better adapting the pre-training weights to the ME datasets. The number of layers in each stage of the two encoders is designed consistent and the loss function is consistent. They can effectively ensure that the output features of each stage of the two-stream networks are at the close level, providing an effective guarantee for feature fusion.

Deep neural networks require a significant amount of data to learn and extract high-level features effectively. However, acquiring ME data is challenging, resulting in limited data availability. When training directly on ME datasets, deep networks are prone to overfitting, which can negatively impact their performance. To address this issue, transfer learning is employed by utilizing pre-trained models trained on the CK+ dataset [31]. Since the tasks of macro expression recognition and MER share similarities, the pre-trained models can be applied to the ME datasets. The two stream networks, which are independent and take optical flow and apex frames as inputs, are pre-trained separately on the corresponding inputs from the CK+ dataset. Specifically, the temporal stream network is pre-trained on the optical flow between the onset frame and apex frame of each sample, while the spatial stream network is pre-trained on the apex frame of each sample.

This step is crucial in the experiment, as it effectively leverages a large number of images from the CK+ dataset. Consistency in the number of layers within each stage of the two encoders and in the loss function ensures that the output features of each stage in the two-stream networks are at a similar level. This provides a reliable foundation for subsequent feature fusion. Furthermore, it is worth noting that optical flow and face images have distinct characteristics. If face images were used for pre-training the two stream networks, a weight mismatch problem would arise, leading to diminished performance. To better adapt the pre-training weights to the ME datasets, the same approach is used to calculate optical flow for both the CK+ dataset and the ME datasets.

Experiments of ablation are designed to illustrate the effectiveness of the proposed transfer learning method, and a full discussion is made in Section 5.

### 3.3. Network Optimization

Cross Entropy loss is utilized as the objective function for training:

(18) $$L=\frac{1}{N}\sum_{i}L_{i}=-\frac{1}{N}\sum_{i}\sum_{c=1}^{C}y_{ic}\log(p_{ic})$$

where *N* represents the number of ME video samples and *C* represents the number of emotion classes, *y_ic_* takes a value of 1 if the true class of sample *i* equals *c*, and 0 otherwise. Similarly, *p_ic_* denotes the predicted probability that sample *i* belongs to class *c*.

We use the Adadelta optimizer [30] to optimize the objective function during network training. This is very necessary for our architecture because it is composed of two completely different cornerstones. When all parameters have a uniform learning rate, it can lead to some parameters changing in the correct direction while others change in the wrong direction. This imbalance can prevent the overall network from converging during training. To address this issue, we utilize the Adadelta optimizer, which adjusts the learning rate based on the parameter’s own change rate. This allows the learning rate to adapt to each parameter individually. In the experiment using gradient descent as the optimizer, we observed that the model failed to converge and could not be effectively trained. Therefore, the use of the Adadelta optimizer with adaptive learning rates is crucial for the successful training and convergence of our model.

It is important to adjust the initial learning rate as the parameters approach their optimum values during training. To achieve this, we utilize cosine annealing [32], which modulates the initial learning rate using a cosine function. This modulation causes the learning rate to decrease slowly at first, then more rapidly, before stabilizing at a lower value. This approach is especially crucial for our current problem, given the limited number of available ME video samples. Without careful handling, this limited dataset can easily lead to overfitting during training. By adjusting the learning rate using cosine annealing, we are able to minimize the risk of overfitting and achieve better generalization performance.

## 4. Experiments

The experiment includes the data used in the experiment, the input of the network, the details of the experiment, and the evaluation metrics.

### 4.1. Datasets

Following the guidelines in the field, we have chosen to utilize three extensive datasets for our assessment. They include the Spontaneous Micro-Expression Corpus (SMIC) [6], the Chinese Academy of Sciences Micro-Expression II data set (CASME II) [33], and the Spontaneous Actions and Micro-Movement database (SAMM) [34]. This selection guarantees a sufficient diversity of data, evaluation scale, and enables fair comparison with other methods in the existing literature.

### 4.2. Inputs

The inputs of the network include the form and the preprocessing method of the inputs.

#### 4.2.1. Long-Term Optical Flow and the Apex Frame

The sequence of ME images is a continuous moving process in time, and each image has a high temporal correlation with its surrounding frames in addition to its own spatial information. In order to extract comprehensive and effective ME features, we extract spatial features from the apex frame of ME and extract temporal features from dense optical flow calculated between the onset frame and apex frame.

#### 4.2.2. Data Pre-Processing

Since the input image of the network should be resized into a shape with the same height and width, in order to prevent image deformation in the process of resizing and maintain the consistency of data between different databases, we adopted the pre-processing method as [2] to pre-process the image sequences in the datasets. First, we employ the Ensemble of Regression Trees [35] algorithm to identify prominent facial landmarks (68 in total) consistently across different datasets for each video sample.

**Figure 8:**
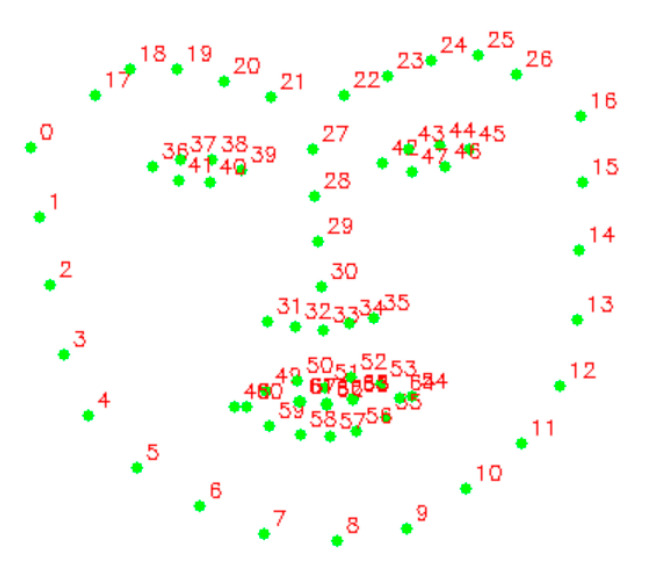
The detected 68 facial landmarks.

As shown in Figure 8, points 27–30 can be used to determine the center line of the nose which can be considered as the vertical symmetry line of the entire face area. Point 30 is set as the center, and the square size s (in pixels) is computed as follows, so that nearly the entire face is included in the cropped image:

(19) $$s=(y_{\left[8\right]}-y_{\left[19\right]})+(y_{\left[8\right]}-y_{\left[57\right]})$$

where $y_{\left[i\right]}$ is the ordinate of point i.

The optical flow captures the amplitude and direction of facial muscle movements, providing important movement trend information. However, if the face is deflected or tilted, the angle information of extracted optical flow will be a superposition of the angle information of both the facial muscle movements and the facial deflection. As the angle information of facial deflection is chaotic, it can interfere with the distinctive dynamic features captured by the optical flow. Therefore, to avoid such interference and maintain the integrity of the distinctive dynamic features, we introduced the rotation alignment operation before cropping the face region. This operation ensured that the facial muscle movements were accurately captured by the optical flow while minimizing any interference caused by head movements, deflection, or tilt.

This rotation alignment operation involved calculating a rotation matrix based on the center coordinates of the eyes and the midpoint of their connection. As shown in Figure 8, we illustrated the process as follows: Firstly, we computed the mean coordinates of points 36–41 to obtain the center coordinates of the left eye, and similarly, we obtained the center coordinates of the right eye using the mean coordinates of points 42–47. Next, we calculated the rotation angle between these two points and the positive direction of the X-axis. Using the OpenCV library, we utilized the *getRotationMatrix2D* function to obtain the affine transformation matrix for rotating the image based on the calculated angle.

**Figure 9:**
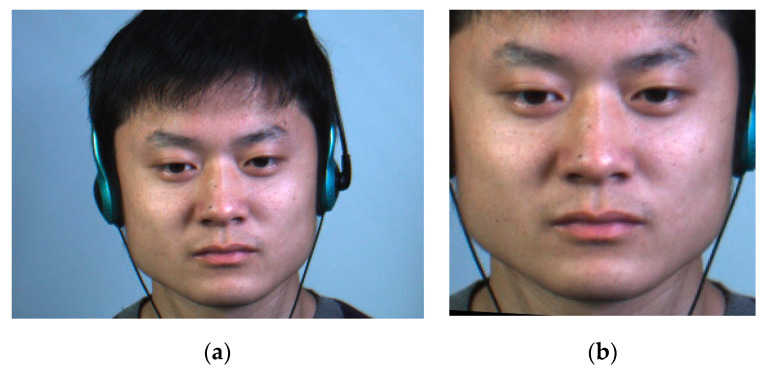
Image changes through preprocessing. (**a**) Raw image; (**b**) Pre-processed image.

We perform facial landmark detection and rotation matrix computation in the apex frame of an ME sample, which is the most informative part of the video. In CASME II and SAMM, the apex frames are explicitly specified, while for SMIC-HS, we select the middle frame of each sample video as the apex frame. Since there are only a few frames available in some samples, we use temporal interpolation in the same way as [2]. After the image is preprocessed, the effect is shown in Figure 9:

### 4.3. Implement Details

In the implementation of transfer learning, we selected the last six emotionally intense images in each sample of video as the input for the spatial stream network. We calculated the optical flows between the onset frame and these frames to serve as input for the temporal stream network. We employed the Haar cascade classifier for the detection and cropping of human faces and then resized the image to 224 × 224. We used cross-entropy loss as the objective function for training. The parameters were set to: epochs 300, batch size 64, and learning rate 0.0001. We adopted an 80% training, and 20% validation split to assess the performance of the two models.

In the temporal feature extraction process, we employed base Swin Transformer blocks, with 12 encoder layers, a hidden size of 48, an MLP size of 3072, and 3, 6, 9, and 12 heads stage by stage. We resized our input optical flow to 224 × 224 pixels and split each image into patches with a size of 4 × 4 pixels, resulting in 56 × 56 patches. 48-dimensional vectors were passed through all Swin Transformer encoder layers.

For the spatial feature extraction process, we employed a convolutional layer and ResNet blocks, with 12 encoder layers, a convolutional kernel size of 3 × 3, and 64, 128, 256, and 512 output channel stages by stage. We resized our corresponding input apex frames to 224 × 224 pixels and passed them through all the ResNet encoder layers.

Regarding the feature fusion algorithm, we carried out experiments on various fusion schemes and verified that the proposed fusion algorithm had the best performance. For temporal aggregation, we first unified the candidate frames into 11 frames near the apex frame through temporal interpolation and then selected five frames (apex, and two preceding and succeeding it) per sample as inputs for the temporal aggregator. In the experiment with LSTM as the aggregator, the input was 11 frames after temporal interpolation.

In the implementation of fine tuning, the parameters were set to: epochs 40, batch size 4, and learning rate 0.003. 

### 4.4. Evaluation Metrics

We carried out experiments on SMIC-HS, CASME II, and SAMM, following previous work and the Micro-Expressions Grand Challenges (MEGCs). The evaluation of classification performance was conducted using a combined corpus created from all three datasets, which was relabeled with three classes according to the proposal in MEGC 2019 [36]. All the findings were obtained through Leave One Subject Out (LOSO) cross-validation. The assessment was conducted repeatedly by withholding test samples from each group of subjects, while the remaining samples were utilized for training. The experiments were carried out on the combined database, comprising three emotional categories (negative, positive, and surprise) obtained from merging SMIC, CASME II, and SAMM databases, totaling 68 subjects with 16 from SMIC, 24 from CASME II, and 28 from SAMM. The performance was evaluated using an unweighted F1-score (UF1), also referred to as macro F1-score, and Unweighted Average Recall (UAR).

(20) UF1=macroF1-score

(21) $$UAR=\frac{\sum_{c=1}^{C}\frac{\sum_{i=1}^{S}TP_{i,c}}{N_{c}}}{C}$$

where Nc is the total number of samples of class c across all subjects, $TP_{i,c}$ represents the true positive for sample *i* of Class *c*.

## 5. Results and Discussion

All optimal results are shown in bold in the following tables.

As shown in Table 3, our approach achieved the best results both in terms of UF1 and UAR on CASME II, on UF1 on SMIC-HS, and UAR on the full composite dataset, and second best on UF1 on the composite dataset, and on UAR on SMIC-HS, and UF1 on SAMM, and on UAR on SAMM. 

Table 4 demonstrates that the feature extraction capability of Swin Transformer greatly exceeds that of ResNet13. By integrating the effective features extracted from both encoders, we were able to improve the network performance substantially.

Table 5 compares the influence of different fusion schemes in our feature fusion algorithm on network performance. The results show that fusion scheme D, which involves no multi-level fusion, has the worst performance. On the other hand, the proposed feature fusion algorithm improves both the F1 score and UAR by approximately 4%, indicating the effectiveness and necessity of the proposed algorithm. In schemes A, B, C, and F, the performance of the network is similar, indicating that the number of stages and the output channel of point convolution are not the primary factors affecting the network performance. To obtain a less complex model, we chose scheme C as the final feature fusion scheme. However, the performance of scheme E is slightly worse. This scheme fuses the output features of stage 2 and stage 4, which are quite different. In the fusion process, 4× upsampling is carried out, while other schemes only process 2× upsampling.

Where Feature Fusion Scheme A~E are as shown in Figure 7, Scheme F changes the number of output channels of point convolution in Scheme C from 256 to 64.

Table 6 compares the impact of different temporal aggregation methods on network performance. Our weighted average operator outperforms the simple mean operator, significantly enhancing network performance on SMIC-HS and CASME II datasets, albeit with less obvious improvements on the SAMM dataset. The reasons for the varied performance are two-fold: Firstly, for the SMIC-HS dataset, we select the middle frame as the apex frame, which may not accurately represent facial expressions at the peak of emotional intensity. Secondly, for the SAMM dataset, all images are grayscale, lacking color information, leading to a diminished distinction between the apex frame and its nearby frames. This is because we select the apex frame and its adjacent 5 frames as the input to the network. The apex frame has the strongest features, which gradually weaken in the neighboring frames. This arrangement allows our weighted average operator to better align with the feature distribution of the input image sequence. However, the performance of LSTM in the network remains poor. This is due to the chaotic facial motion near the apex frame, where temporal features are less prominent, making it challenging for LSTM to capture meaningful patterns in the data.

Table 7 compares the impact of different input configurations on the performance of transfer learning in the temporal stream network. In both the pre-training and fine-tuning stages, the input configuration of the temporal stream network remains consistent. Specifically, the optical flow between the onset frame and apex frame is used as the input, which yields better results compared to using face images as input. This choice is motivated by the distinct distribution of optical flow and face images. By utilizing optical flow as input, the pre-trained model’s weights align more closely with the input during the fine-tuning process. This improves the compatibility between the model and the data, leading to enhanced performance in transfer learning. This method mainly works well on CASME II, probably because the distribution of optical flow in the dataset used for pre-training is more consistent with the distribution of optical flow in CASME II.

Table 8 demonstrates that our model exhibits significantly shorter inference times for four video samples compared to SLSTT-LSTM. This improved efficiency can be attributed to the architectural choices we made in our two-stream model. Specifically, our temporal encoder utilizes the Swin Transformer architecture, which reduces computational requirements when compared to the ViT architecture. Similarly, our spatial encoder employs the ResNet architecture, which also requires less computation.

Compared to SLSTT, our approach incorporates a spatial encoder to extract face texture features, effectively enhancing the feature representation. By utilizing the Swin Transformer architecture, we are able to capture facial muscle movement features from the optical flow, surpassing the performance of the Vision Transformer used in SLSTT. Additionally, the proposed feature fusion algorithm, weighted average operator, and transfer learning method further contribute to the overall performance improvement. These enhancements collectively enable our model to achieve better results in MER.

Although we use the widely used spontaneous ME datasets to verify the proposed method, so as to increase the applicability of the method in the real world as much as possible, after all, these datasets are obtained under strict experimental conditions, and there is a gap between them and the real world. In the field forum, some experts also mentioned that the application of these existing methods to the real world is not very good, and requires more extensive and in-depth research. However, our proposed method can provide references for real-world applications, especially feature fusion algorithms and training methods, which do improve performance on these datasets.

## 6. Conclusions

In this paper, we propose a novel spatio-temporal deep learning architecture for micro-expression recognition, which combines transformer-based Swin Transformer with CNN-based ResNet at multiple feature levels. Our architecture enables simultaneous learning of local and global relationships within the window of optical flow, relationships between windows, and texture features in the spatial dimension. Furthermore, it reduces computational requirements compared to ViT-based architecture. Unlike traditional two-stream architectures that rely on late fusion and only make use of output features at the deepest level of the network, the proposed feature fusion algorithm leverages the similarity of output feature shapes at each stage of the two encoders. This allows us to realize the fusion of multi-level spatio-temporal features and improve both the F1 score and UAR by approximately 4%. Additionally, the proposed weighted average operator takes advantage of the feature distribution of the input image sequence, surpassing the simple mean operator. We emphasize the importance of maintaining consistent input form for the same encoder during pre-training and fine-tuning, as well as selecting the appropriate optimizer based on the architectural characteristics. Through extensive experiments using three large MER databases with composite database evaluation setups and Leave-One-Subject-Out cross-validation protocols, our method consistently outperforms other comparative methods.

One limitation of our work is the cumbersome training process. During the processes of pre-training and fine-tuning, it is necessary to maintain consistency in the input format and optical flow extraction method. Furthermore, our proposed method, like many others in the field, relies on apex frames for MER. However, it does not address the integration of apex frame detection and the recognition network, which could potentially limit its performance in certain scenarios. These limitations suggest areas for future research, such as exploring more efficient training strategies and investigating methods that better integrate apex frame detection with the recognition network.

## Figures and Tables

**Table 1:** Detailed specifications of ResNet13 architecture.

Layer	Parameters	Output Size
Conv1	3 × 3 S2 K64	112 × 112
Pool1	2 × 2	56 × 56
Stage 1	$\left[\begin{array}{c} 3\times 3,\;K64\\ 3\times 3,\;K64 \end{array}\right]$ × 2	56 × 56
Stage 2	$\left[\begin{array}{c} 3\times 3,\;K128\\ 3\times 3,\;K128 \end{array}\right]$ × 2	28 × 28
Stage 3	$\left[\begin{array}{c} 3\times 3,\;K256\\ 3\times 3,\;K256 \end{array}\right]$ × 2	14 × 14
Stage 4	$\left[\begin{array}{c} 3\times 3,\;K512\\ 3\times 3,\;K512 \end{array}\right]$ × 2	7 × 7

**Table 2:** Detailed specifications of Swin Transformer architecture.

	Downsp. Rate (Output Size)	Swin Transformer
Stage 1	4× (56 × 56)	Concat 4 × 4 96-d, LN
		$\left[\begin{array}{c} \mathrm{Win}.\;\mathrm{Sz}.\;7\times 7,\\ \dim\;96,\;\mathrm{head}\;3 \end{array}\right]$ × 2
Stage 2	8× (28 × 28)	Concat 2 × 2 192-d, LN
		$\left[\begin{array}{c} \mathrm{Win}.\;\mathrm{Sz}.\;7\times 7,\\ \dim\;128,\;\mathrm{head}\;6 \end{array}\right]$ × 2
Stage 3	16× (14 × 14)	Concat 2 × 2 384-d, LN
		$\left[\begin{array}{c} \mathrm{Win}.\;\mathrm{Sz}.\;7\times 7,\\ \dim\;256,\;\mathrm{head}\;12 \end{array}\right]$ × 6
Stage 4	32× (7 × 7)	Concat 2 × 2 768-d, LN
		$\left[\begin{array}{c} \mathrm{Win}.\;\mathrm{Sz}.\;7\times 7,\\ \dim\;512,\;\mathrm{head}\;24 \end{array}\right]$ × 2

**Table 3:** CDE results comparison with LOSO on SMIC-HS, CASME II, SAMM, and Composed Database (3 classes).

Method	Composite		SMIC-HS		CASME II		SAMM	
	UF1	UAR	UF1	UAR	UF1	UAR	UF1	UAR
LBP-TOP	0.588	0.579	0.200	0.528	0.703	0.743	0.395	0.410
Bi-WOOF	0.630	0.623	0.573	0.583	0.781	0.803	0.521	0.514
ResNet18	0.589	0.563	0.463	0.433	0.625	0.614	0.476	0.436
DenseNet121	0.425	0.341	0.460	0.333	0.291	0.352	0.565	0.337
Inception V3	0.516	0.504	0.411	0.401	0.589	0.562	0.414	0.404
WideResNet28-2	0.505	0.513	0.410	0.401	0.559	0.569	0.410	0.404
OFF-ApexNet [37] (2019)	0.720	0.710	0.682	0.670	0.876	0.868	0.541	0.539
CapsuleNet [19] (2019)	0.652	0.651	0.582	0.588	0.707	0.701	0.621	0.599
Dual-Inception [38] (2019)	0.732	0.728	0.665	0.673	0.862	0.856	0.587	0.566
STSTNet [39] (2019)	0.735	0.761	0.680	0.701	0.838	0.869	0.659	0.681
ATNet [40] (2019)	0.631	0.613	0.553	0.543	0.798	0.775	0.496	0.482
RCN [41] (2020)	0.705	0.716	0.598	0.599	0.809	0.856	0.677	**0.698**
SLSTT-LSTM [2] (2022)	**0.816**	0.790	0.740	**0.720**	0.901	0.885	**0.715**	0.643
TFT (Ours)	0.814	**0.801**	**0.741**	0.718	**0.907**	**0.909**	0.709	0.656

**Table 4:** Ablation Study of the Two-Stream Architecture.

Architecture	Composite		SMIC-HS		CASME II		SAMM	
	UF1	UAR	UF1	UAR	UF1	UAR	UF1	UAR
Swin_Transformer	0.655	0.639	0.623	0.622	0.779	0.762	0.592	0.581
ResNet13	0.437	0.438	0.413	0.425	0.465	0.475	0.415	0.413
Swin_Transformer+ ResNet13	**0.717**	**0.694**	**0.645**	**0.637**	**0.829**	**0.815**	**0.633**	**0.586**

**Table 5:** Ablation Study of the Feature Fusion Algorithm.

Feature Fusion Scheme	Composite		SMIC-HS		CASME II		SAMM	
	UF1	UAR	UF1	UAR	UF1	UAR	UF1	UAR
A	0.808	0.798	0.736	0.709	0.896	0.888	0.703	**0.658**
B	0.809	0.796	0.738	0.716	0.897	0.892	0.702	0.653
C	**0.814**	**0.801**	**0.741**	**0.718**	**0.907**	**0.909**	**0.709**	0.656
D	0.773	0.754	0.706	0.694	0.859	0.845	0.676	0.593
E	0.798	0.782	0.721	0.686	0.892	0.882	0.687	0.635
F	0.809	0.793	0.732	0.706	0.900	0.889	0.698	0.642

**Table 6:** Ablation Study of Temporal Aggregation.

Temporal Aggregation Method	Composite		SMIC-HS		CASME II		SAMM	
	UF1	UAR	UF1	UAR	UF1	UAR	UF1	UAR
Mean Operator	0.791	0.779	0.718	0.704	0.868	0.855	0.692	0.642
Weighted Average Operator	**0.814**	**0.801**	**0.741**	**0.718**	**0.907**	**0.909**	**0.709**	**0.656**
LSTM Aggregator	0.545	0.540	0.471	0.518	0.478	0.480	0.581	0.569

**Table 7:** Ablation Study of the different inputs of a temporal encoder in Transfer Learning.

Temporal Stream Network	Composite		SMIC-HS		CASME II		SAMM	
	UF1	UAR	UF1	UAR	UF1	UAR	UF1	UAR
Pre-trained on raw image	0.806	0.783	**0.744**	**0.720**	0.865	0.857	**0.711**	**0.658**
Pre-trained on optical flow	**0.814**	**0.801**	0.741	0.718	**0.907**	**0.909**	0.709	0.656

**Table 8:** Inference Time of Model for four video samples on the same device.

Model	Time (s)
SLSTT-LSTM	2.363
TFT (Ours)	1.423

## Data Availability

We will publish the core code on the GitHub website: https://github.com/wzb0718/My-code.
